# Patterns and temporal trends in canine breakage and scarring in Western Hudson Bay polar bears (*Ursus maritimus*)

**DOI:** 10.1371/journal.pone.0319753

**Published:** 2025-03-25

**Authors:** Simonne S. Tremblay, David McGeachy, Nicholas J. Lunn, Evan S. Richardson, Andrew E. Derocher

**Affiliations:** 1 Department of Biological Sciences, University of Alberta, Edmonton, Alberta, Canada; 2 Wildlife Research Division, Science & Technology Branch, Environment and Climate Change Canada, University of Alberta, Edmonton, Alberta, Canada; 3 Wildlife Research Division, Science and Technology Branch, Environment and Climate Change Canada, Winnipeg, Manitoba, Canada; Liverpool John Moores University, UNITED KINGDOM OF GREAT BRITAIN AND NORTHERN IRELAND

## Abstract

Canines are used by carnivores for prey capture and social interactions but are often damaged. The highly carnivorous polar bear (*Ursus maritimus*) has a female defence polygyny mating system where males compete for access to females and injuries to males, such as broken canines and cuts, are common. The Western Hudson Bay polar bear subpopulation has declined in abundance in recent decades and shifted from a female-biased to a male-biased adult sex ratio, which may have affected their mating system. We hypothesize that if changes in subpopulation structure have affected the mating system, then canine breakage and scarring may have changed over time. We assessed age- and sex-specific occurrences of canine breakage and scarring in 3493 individuals between 1981-2023 using non-parametric statistical analyses and linear mixed effect models. We found age- and sex-related differences in mean values of breakage and scarring. These injury occurrences increased with age in both sexes and males had greater amounts of both breakage and scarring compared to females. As the only main effect, sampling year was significant and indicated increasing breakage in both sexes over time; however, the top breakage model did not include year, indicating it was not as strong a predictor of breakage and scarring as age or sex. Age, sex, and year were all in the top model for predicting maximum scarring probabilities. We found some evidence that injuries changed over time, suggesting there could be changes to intraspecific interactions, but additional monitoring is needed.

## Introduction

Specializations in heterodont dentition are numerous across the Carnivora and reflect their diverse life histories and specializations [1–4]. Carnivores typically use anterior teeth for prey capture, social displays, and interactive behaviours, while food processing is done primarily by their carnassial teeth [1–3]. Canines are characterized by their long-pointed conical shape that vary in thickness, curvature, and length based on species-specific feeding ecology [1,4]. Despite their importance in predatory behaviours and day-to-day interactions, canines have the highest incidence of breakage among carnivorous mammals compared to other tooth types [4,5]. Loss of canine function can affect hunting ability, feeding, and defence, which may influence an individual’s fitness and survival [5–7]. Tooth breakage occurs once the biomechanical limitations of the canines are exceeded and is often associated with prey acquisition but can also occur during aggressive encounters separate from feeding [4,5,7]. Intersexual behavioural differences may also affect canine breakage [5,8,9]. Therefore, canine breakage can provide insights into aspects of life history such as dietary shifts [10], age- and sex-related behavioural differences [5,8,11], and competition [8,12].

The eight Ursidae species are typically large, have a muscular build, solitary lifestyles, occupy a wide range of habitats, and fill diverse ecological niches [1,13,14]. Despite some ursids adapting to specialized feeding ecologies [15,16], all exploit an omnivorous diet to varying degrees [1,13]. This generalist diet is reflected in ursid dentition through less derived tooth morphologies than other Carnivora such as canids or felids [2,17,18]. Ursids with more predatory niches, such as the hyper-carnivorous polar bear (*Ursus maritimus*) and its omnivorous sister taxa the brown bear (*U. arctos*), have only minor adaptations for a carnivorous diet [17]. The lack of craniodental specialization for such a diet in these two species may be associated with the role of strength during predation, thus reducing selection for highly adapted tooth morphologies [17–19]. Similar to other carnivores, ursids experience canine breakage and other tooth injuries, despite having less specialized anterior dentition [20–22]. Intersexual differences in canine breakage also occur in ursids, exemplified by male polar bears experiencing greater breakage than females [8,22,23].

Polar bears are an apex predator of ice-covered Arctic marine ecosystems and the largest of the Ursidae [1,13,16]. They have adapted to Arctic environments and a predatory niche following their divergence from brown bears by specializations including white pelage, altered skull morphology, sharp and curved claws, and appendages modified for aquatic locomotion [1,16,24,25]. Sexual dimorphism is common in ursids and is particularly pronounced in polar bears with one of the greatest intersexual differences in mammals [13,26]. Although the most sexually dimorphic character is body mass, with adult males being about twice the size of adult females, other differences include foreleg guard hair length, skull size, and body length [26]. Dentition also differs with males possessing longer molar rows [27] and more robust canines [28] (S1 Fig).

The interbirth interval is typically three years for polar bears because of extended cub dependency on their mothers [29,30]. As a result, polar bear populations show skewed operational sex ratios (OSR) as there are more males able to mate than available females [8]. This sex ratio asymmetry can lead to a female defence polygynous mating system where males compete for access to breeding females [23,31,32]. The mating system of polar bears, however, has also been defined as serial monogamy [23], polyandry [8], and promiscuity [33] depending on the temporal perspective. Male polar bears search for and may fight for access to breeding females [23]. Correlated traits such as larger size and prime age (i.e., mid-teens) are associated with higher mating success [32,34]. Intrasexual combat among male polar bears often results in injuries with varying severity, most commonly canine breakage and/or wounds that will scar, which suggests these interactions are intense and may reduce survival by decreasing their ability to effectively capture prey and defend themselves following injury [8,23]. Therefore, mating-related injuries assessed at a subpopulation level may provide insights into intraspecific competition and possibly as a response to shifting demographics.

Polar bears have a circumpolar Arctic distribution in 20 subpopulations [35,36]. The Western Hudson Bay (WH) subpopulation is characterized as a seasonal ice ecoregion as bears are forced onshore without access to prey for portions of the year once the sea ice melts in Hudson Bay [37,38]. This subpopulation has been the subject of long-term studies since the early 1980s, which has allowed insights into population dynamics that are often unavailable elsewhere [39,40]. Negative effects of climate warming, notably shifts in dates of ice formation and breakup that extend the period spent ashore, have been documented, resulting in decreased recruitment and survival rates, lower body condition, and a decline in abundance [39,41,42]. The WH subpopulation has shifted from a female-biased adult sex ratio [43] to a possibly male-biased adult sex ratio [36] through population decline and lower female survival [36,39]. Male competition may intensify if female availability declines [31] and, therefore, an increase in male abundance could further skew the already male-biased OSR and influence the mating system.

We investigated patterns and temporal trends of intraspecific injuries in WH polar bears using data collected from free-ranging bears from 1981 to 2023 to: 1) assess age- and sex-specific patterns of canine breakage and scars, and 2) examine temporal trends in canine breakage and scar occurrences. We hypothesized that changes in sex ratio towards males may have increased competition for mates and thus increased canine breakage and scarring over time.

## Materials and methods

### Study area

**Fig 1:**
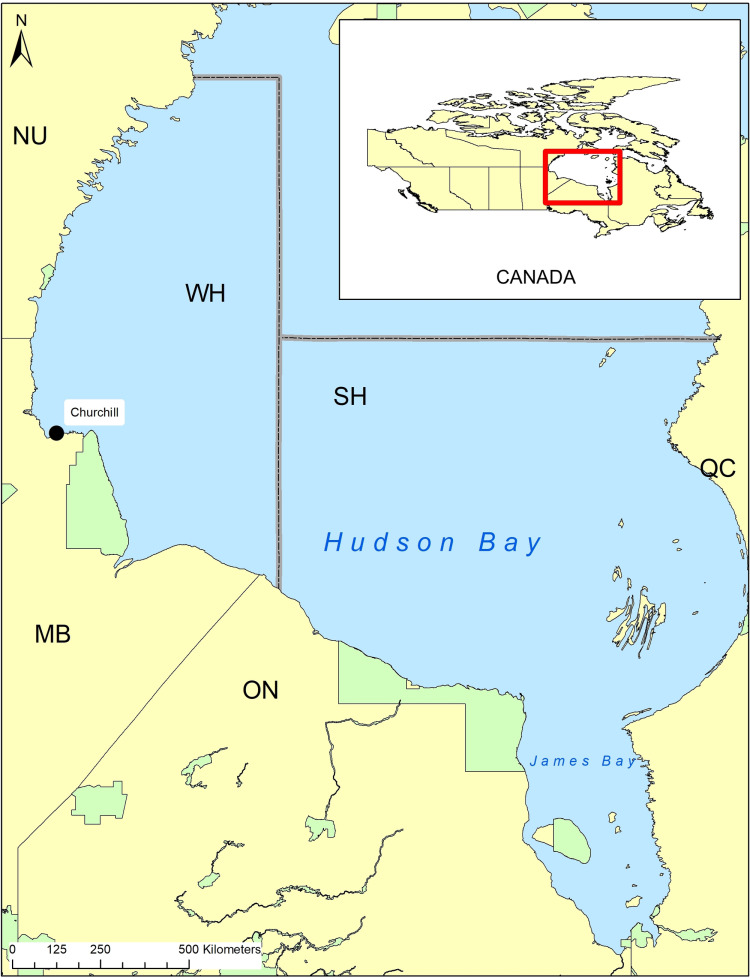
Map of Western Hudson Bay, Canada. Subpopulation boundaries are represented by the dotted grey line, provided by Environment and Climate Change Canada. Green polygons denote parks and protected areas within the region. Basemap, provincial, and territory boundaries were retrieved from Statistics Canada 2021 (https://www12.statcan.gc.ca/census-recensement/2021/geo/sip-pis/index-eng.cfm).

The boundaries of the WH subpopulation fall within western Hudson Bay in northern Canada, encompassing terrestrial areas in northeastern Manitoba, southern Nunavut, and northwestern Ontario and adjacent seasonal sea ice offshore (Fig 1). Hudson Bay is a shallow marine environment (largely < 200 m deep) and has a colder regional climate than typical at the respective latitude [44]. Terrestrial areas are part of the Hudson Bay Lowlands and are characterized as cold wetlands and tundra near the coast and form an ecotone with boreal forest further inland [45,46]. Polar bears within WH exhibit high site fidelity and are largely separated from adjacent subpopulations during ice-free periods despite mixing on the sea ice [47,48].

### Capture and handling

**Fig 2:**
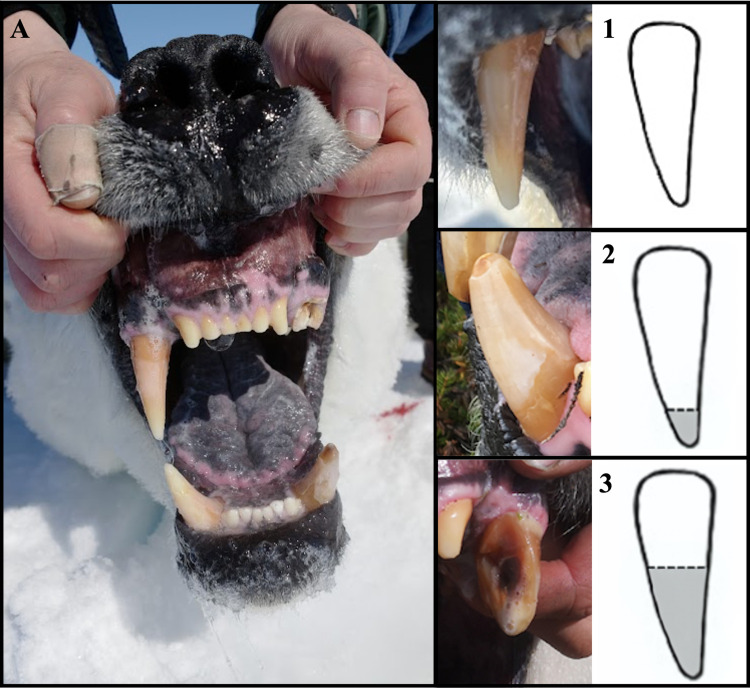
Canine breakage categories. **(A)** Canine classifications (starting top left clockwise): 1, 3, 2, 1. Side panels depict illustrative approximate vertical cut-offs for each breakage classification and real-life examples associated with each category, denoted by number. Images taken by David McGeachy and Andrew E. Derocher. Image in Panel A reprinted from [52] under a CC BY license, with permission from Andrew E. Derocher, original copyright [2023]. Illustrations by Simonne S. Tremblay.

WH polar bears were opportunistically sampled in July to November from 1981 to 2023 excluding 2020 throughout the terrestrial study area (Fig 1) following standardized chemical immobilization capture techniques [49]. Body morphometrics were standardized for consistency across observers [50]. Sex and reproductive status were recorded, and a vestigial premolar was extracted to estimate age for bears ≥ 2 years old [51]. Bears < 2 years old were aged using tooth eruption patterns [51]. Both sexes were assessed for canine breakage with damage to each canine quantified as follows: 1 (minimal to none), 2 (moderate), and 3 (excessive) (Fig 2) and align with past studies [8,23,32]. Additional examples of canine breakage are available as supplementary material (S2 Fig). For brevity, canine breakage will be referred to as “breakage”. Scarring was similarly classified from 1 to 3 based on the number and severity [23,32]. Records of individuals sampled after handling by the Government of Manitoba were excluded to remove possible confounding breakage as bears held in the Churchill Polar Bear Holding Facility [53,54] can break their teeth in captivity.

Animal handling was conducted under annual animal care approvals from Environment and Climate Change Canada's Western and Northern Animal Care Committee (2001PNR013, 2002PNR013, 2003PNR013, 2004PNR013, 2005PNR013, 2006PNR013, EC-PN-07-013, EC-PN-08-013, EC-PN-09-013, EC-PN-10-013, EC-PN-11-013, EC-PN-12-013, EC-PN-13-013, EC-PN-14-013, 15NL01, 16NL01, 17NL01, 18NL01, 19NL01, 21NL01, 22NL01, 23ER01) and from the University of Alberta Animal Care Committee (Animal Use Protocols 00000033 and 00003667) consistent with the Canadian Council on Animal Care. Research permits were issued by Parks Canada Agency and the Province of Manitoba.

### Statistical analyses

Temporal trends in mean age were assessed by linear regression using year as the predictor; mean age was regressed using a log transformation to improve normality then transformed back to the original scale for interpretation. We assessed error rates in canine classifications to determine repeatability using the mean breakage averaged across the canines, which should remain constant or increase over an individual’s life. Therefore, resampled individuals were assessed to determine if their mean breakage observation ever decreased when recaptured.

Normality in breakage and scarring data were assessed using histograms, Kolmogorov-Smirnov tests and Shapiro-Wilks tests; log transformations did not improve deviations from normality, so all analyses were non-parametric. We assessed differences in breakage values between the four canines in both sexes using Kruskal-Wallis chi-squared tests in lieu of a one-way analysis of variance [55]; *post-hoc* analysis used a Dunn’s test of multiple comparisons with a Bonferroni adjusted p-value [56,57]. Comparison of intersexual mean breakage used an unpaired Wilcoxon test. We performed an analysis of covariance (ANCOVA) using a linear mixed effects model where age and sex of the individuals were the fixed-effect predictors on mean breakage while controlling for the random effects of repeated individuals. An interaction between these predictors was tested in the ANCOVA. Scars were assessed using a McNemar’s test, a non-parametric 2x2 chi-squared equivalent [58], comparing the presence of scars as a factor of breakage presence in both males and females. Presence of breakage represents a binary variable quantifying whether individuals had breakage on any measured canine. Similarly, presence of scars dichotomously classified individuals on whether they had scars or not.

Temporal trends in breakage were examined through additive and interactive linear mixed effects models. Using the fixed predictors of year and age, while controlling for individual as a random effect, we regressed on mean breakage for each sex. Significance of predictors and outputs helped determine whether additive or interactive models more appropriately described temporal trends of breakage. A full model using age, sex, and year as fixed predictors and individual as the random predictor was also constructed to test the overall main effects, first-order interactions, and second-order interaction. All breakage models were fit using restricted maximum likelihood. Scarring temporal trends were examined using additive mixed multinomial logistic regressions using the fixed predictors of age, sex, and year. While controlling for the random effects of individual, all possible combinations of the main effects were assessed as significant predictors in maximum scar value. Maximum scar values represent the highest recorded value of an individual’s observed scars, ranging from 1 to 3 with 1 being the reference level of no wounds. Model selection for breakage temporal trends was performed using the dredge function in R’s *MuMIn* package, while scarring’s temporal model selection was performed manually; both analyses used Akaike’s information criterion (AIC) values for model ranking, with models considered higher ranked from the next model if ΔAIC was >  2 [59].

All analyses were completed using individuals from all age classes. Statistical procedures were performed in Microsoft Excel and R version 2023.12.0 + 369 [60]. Means are presented with ±  standard error. Statistical significance was p ≤  0.05 and marginal significance for p-values between 0.051 to 0.1. For linear mixed models, conditional and marginal R^2^ values are provided, and all top models are presented with standardized regression coefficients to illustrate effects of each predictor. This study’s data is publicly available for review and use at the University of Alberta Dataverse in Borealis: The Canadian Dataverse Repository (https://doi.org/10.5683/SP3/SYFUPL).

## Results

Over the study period, a total of 3493 bears were captured with a mean of 84 bears/year ( ± 8 bears, range 3-228), including 1921 females (46 ±  5 females/year, range 0-127) and 1572 males (38 ±  4 males/year, range 1-101). The mean age was 9.6 years ( ± 0.3 years, range 0-30, n =  3493) and age increased over time (linear regression: y =  0.0487 +  1.0020year; F_1,40_ =  4.25, p =  0.046). Our dataset included 812 individuals with repeated captures and changes in classification were uncommon with only 4.1% (33/812) of mean canine classifications being lower on subsequent captures (mean =  0.40 ±  0.04 decrease, range =  0.25-1.25, n = 33).

### Canine breakage and scarring patterns

**Fig 3:**
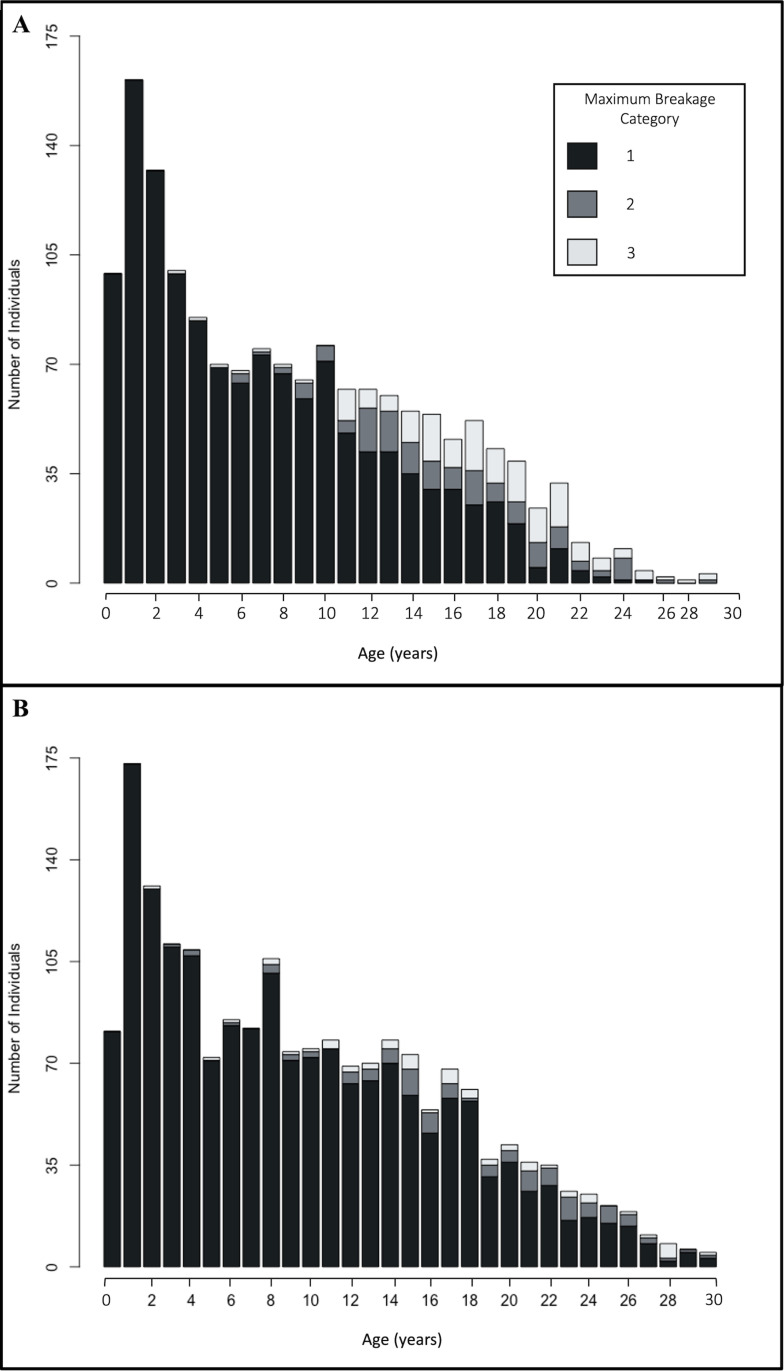
Maximum breakage occurrence in Western Hudson Bay polar bears by age, 1981-2023. (A) males, **n** =  1572. (B) females, **n** =  1921. Maximum breakage represents the greatest value of canine breakage across an individual’s canines.

Breakage in males varied significantly among the four canines (Kruskal-Wallis test: H_3_ =  17.56, p =  0.0005). Pairwise comparisons indicated that breakage was significantly different in males for lower left versus upper right (Dunn’s test: p =  0.0005) and lower right versus upper right canines (Dunn’s test: p =  0.015). The lower left canine had the highest mean breakage in males (Table 1). In females, breakage varied with marginal significance between canines (Kruskal-Wallis test: H_3_ =  6.57, p =  0.087). Severe breakage in both canines of the same jaw was uncommon in both sexes but more frequent in males (both upper canines =  3: males =  1.34% (21/1572), females =  0.05% (1/1971); both lower canines =  3: males =  1.59% (25/1572), females =  0.10% (2/1921)). Mean breakage differed between the sexes with males having greater mean values than females (Wilcoxon rank sum test: W =  9788029, p <  0.0001). Age, sex, and the interaction between the two variables were all significant predictors for mean breakage (ANCOVA: y =  0.9813 +  0.0051age – 0.0617sex +  0.0170age*sex; p_age_ <  0.0001, p_sex_ <  0.0001, p_age*sex_ <  0.0001, conditional R^2^ =  0.430, marginal R^2^ =  0.254). Maximum breakage was similar between the sexes during the first ten years of life (males: 1.03 ±  0.007 mean maximum breakage, range 1-3, n =  1001; females: 1.02 ±  0.006 mean maximum breakage, range 1-3, n =  1099). However, males accrued higher breakage around 11 years old and more individuals had higher maximum breakage in their later years (Fig 3A). The number of females with higher canine classifications also increased with age but not at the same frequency as males (Fig 3B).

**Table 1:** Mean breakage value in each canine tooth in Western Hudson Bay polar bears, 1981-2023.

Tooth Position	Males^a^	Females^a^
Upper left	1.08 ( ± 0.01)	1.03 ( ± 0.004)
Upper right	1.07 ( ± 0.01)	1.02 ( ± 0.003)
Lower left	1.12 ( ± 0.01)	1.05 ( ± 0.01)
Lower right	1.11 ( ± 0.01)	1.03 ( ± 0.005)

^a^Standard errors of means are expressed in brackets.

For both sexes, the proportion of individuals with scars significantly differed based on the presence of breakage (males: McNemar’s test: X^2^ =  51.70, df =  1, p <  0.0001; females: McNemar’s test: X^2^ =  39.70, df =  1, p <  0.0001). Individuals with breakage had more scarring when compared to those with no breakage. The proportion of individuals with scars but no breakage was similar between the sexes (Tables 2 and 3). However, the proportions of males with scars and breakage (Table 2), was nearly double that of females with both scars and breakage (Table 3).

**Table 2:** Proportions of scarring in male polar bears based on the presence of canine breakage in Western Hudson Bay polar bears.

Injury Combination	No Breakage^a^	Breakage^a^
No Scars	1065 (91.3%)	235 (58.0%)
Scars	102 (8.7%)	170 (42.0%)
Total per Breakage Category	1167 (100%)	405 (100%)

^a^Percentage of individuals in each breakage provided in brackets.

**Table 3:** Proportions of scarring based on the presence of canine breakage in female polar bears in the Western Hudson Bay.

Injury Combination	No Breakage^a^	Breakage^a^
No Scars	1726 (92.8%)	48 (78.7%)
Scars	134 (7.2%)	13 (21.3%)
Total per Breakage Category	1860 (100%)	61 (100%)

^a^Percentage of individuals in each category provided in brackets.

### Temporal trends

Year was significant in predicting male mean breakage when it was the only fixed effect (linear mixed effect model: y =  -6.495 +  0.0038year, p_year_ <  0.0001, conditional R^2^ =  0.311, marginal R^2^ =  0.022) with an increase in breakage over time. Year was marginally significant in the male additive model for year and age (linear mixed effect model: y =  -1.237 +  0.0011year +  0.0218age, p_year_ =  0.080, p_age_ <  0.0001, conditional R^2^ =  0.491, marginal R^2^ =  0.253). In the interactive model, age and the interaction term were significant predictors, while year was marginally significant (linear mixed effect model: y =  4.577 −  0.0018year −  0.6126age +  0.0003year*age, p_year_ =  0.071, p_age_ =  0.0005, p_year*age_ =  0.0003, conditional R^2^ =  0.492, marginal R^2^ =  0.254). Similar to males, year was a significant predictor with an increasing trend in female mean breakage as the only predictor (linear mixed effect model: y =  -0.8397 +  0.0009year, p_year_ =  0.0007, conditional R^2^ =  0.324, marginal R^2^ =  0.006) and became marginally significant when age was added to the model (linear mixed effect model: y =  -0.0019 +  0.0005year +  0.0049age, p_year_ =  0.065, p_age_ <  0.0001, conditional R^2^ =  0.358, marginal R^2^ =  0.066). The interactive model found all three variables to be significant when predicting female temporal mean breakage (linear mixed effect model: y =  3.265 −  0.0011year −  0.3277age −  0.0002year*age, p_year_ =  0.011, p_age_ <  0.0001, p_year*age_ <  0.0001, conditional R^2^ =  0.364, marginal R^2^ =  0.078). In the full model evaluation, the top model included the fixed predictors age and sex and the first order interactive term of age*sex (Table 4; conditional R^2^ =  0.430, marginal R^2^ =  0.254, standardised regression coefficient & 95% CI: Intercept =  -0.16 [-0.21, -0.12], Age =  0.17 [0.13, 0.21], Sex =  0.45 [0.38, 0.52], Age*Sex =  0.56 [0.50, 0.62]). In the next two top models, all three predictors were included and contained varying first-order interactions, but none of the top three models included the three-way interaction term: age*sex*year (Table 4).

**Table 4:** Top three linear mixed effect models for mean breakage prediction in Western Hudson Bay polar bears from 1981-2023 based on Akaike information criterion (AIC).

Model Rank	Predictors^a^	Log-likelihood	AIC	ΔAIC
1	Age, Sex, Age*Sex	1008.76	-2005.20	0.00
2	Age, Sex, Year, Age*Sex, Age*Year	1008.68	-2001.40	3.80
3	Age, Sex, Year, Age*Sex	1004.35	-1994.65	10.45

^a^Fixed effect predictors include age of individual, sex, and year, with the models testing main effects, first-order interactions between the main effects, and three-way interaction between main effects. Random effect predictor was individual.

For the temporal trends in scars, the top model for maximum scarring probabilities included all three main effects of age, sex, and year (multinomial logistic regression: rank 1_age_: =  0.23, SE =  0.01, 95% CI =  [0.21, 0.26], p <  0.0001, standardised regression coefficient & 95% CI =  1.64 [1.47, 1.81]; rank 2_age_: =  0.34, SE =  0.02, 95% CI =  [0.29, 0.38], p <  0.0001, standardised regression coefficient & 95% CI =  2.37 [2.06, 2.68]; rank 1_sex_: =  3.62, SE =  0.20, 95% CI =  [3.22, 4.01], p <  0.0001, standardised regression coefficient & 95% CI =  3.61 [3.22, 4.01]; rank 2_sex_: =  4.53, SE =  0.36, 95% CI =  [3.82, 5.24], p <  0.0001, standardised regression coefficient & 95% CI =  4.53 [3.82, 5.24]; rank 1_year_: =  0.02, SE =  0.01, 95% CI =  [0.01, 0.03], p =  0.004, standardised regression coefficient & 95% CI =  0.18 [0.06, 0.31]; rank 2_year_: =  -0.01, SE =  0.01, 95% CI =  [0.03, 0.01], p =  NA, standardised regression coefficient & 95% CI =  -0.10 [-0.31, 0.11]) (Table 5). Hauck-Donner effects [61] were detected in some intercepts and ranks of each model tested, including the top model’s rank1_intercept_ and rank2_year_, hence the omission of p-value estimates for afflicted coefficients. Despite these effects, we believe the top model was valid based on robust confidence intervals of all other coefficients and AIC performance with respect to the other models.

**Table 5:** Top three mixed multinomial logistic regression models for maximum scar value prediction in Western Hudson Bay polar bears from 1981-2023 based on Akaike information criterion (AIC).

Model Rank	Predictors^a^	Log-likelihood	AIC	ΔAIC
1	Age, Sex, Year	-1041.90	2099.79	0.00
2	Age, Sex	-1047.83	2107.65	7.86
3	Age, Year	-1386.67	2785.34	685.55

^a^Main fixed effect predictors include age of individual, sex, and year. Random effect predictor was individual.

We also examined temporal trends using only adult bears (older than four years of age) in our analyses. We found similar results to those presented above, with both breakage and scarring’s top models remaining the same (S1 File).

## Discussion

Age and sex were the strongest correlates with breakage in WH polar bears, breakage increased with age in both sexes and males had greater overall breakage. These findings align with past studies in polar bears [22,23,32]. Similar age- and sex-related patterns have been noted in wolves (*Canis lupus*) [5,62]. If population structure changes influenced competition for mates, which in turn influenced breakage or scarring, we may have lacked statistical power to detect such temporal trends. On its own, year was a significant predictor for mean breakage within WH bears. However, it was not significant when age, sex, and interactive effects were added to the model and was not included as an effect in the top model. In the predictions for scarring probabilities, the top model produced included year as a main effect. Together, these results suggest there might be some temporal influence on intraspecific injuries, and thus competition, in WH polar bears, but its importance has variable significance and little clarity at this time. We found that the mean age of bears increased over the study, with a + 0.2% rate of change per year, aligning with other recent WH studies [63]. Given our findings establish a relationship between age and injury in WH bears, this age-related trend may be the driver behind the possible temporal trends observed. Regardless, age and sex were found to have a stronger relationship to breakage and scarring than any possible temporal aspects.

Our study has some limitations. Based on the opportunistic capture design, we do not know if breakage affects survival. Previous studies have established an association between increased tooth wear and lower survival in mammals [64,65]. If mortality is increased by canine damage, this could reduce the number of individuals with higher breakage in the population and our sample. Further, we assume that subpopulation adult sex ratios observed on land in aerial surveys reflect sex ratios on sea ice during mating season. However, WH bears can overlap with individuals from the Southern Hudson Bay and Foxe Basin subpopulations during the mating season [48,66] and if these subpopulation sex ratios were different, we may not have an estimate of the OSR WH bears were exposed to during mating. High recapture rates for individuals in the WH could also present a source of bias as injury scores should only remain the same or increase, potentially leading observers to overscore a breakage or scarring value. While many of recaptured individuals maintained their previous score throughout capture history, this is an important potential source of error to acknowledge. Additionally, given the nature of breakage classification, the metric is unlikely to be overly powerful for detecting temporal trends. Lastly, sampling may not be long enough to assess possible changes in breakage after the shifts in WH demographics. Patterns from population change can take decades to appear [67]; and while we found minor suggestions of temporal change in injuries, further monitoring is warranted to increase statistical power.

We found the lower left canines had greater breakage values than the other teeth in males, while females had a marginally significant difference in breakage among canines. This pattern was similar to raccoon dogs (*Nyctereutes procyonoides*), which had greater dental pathologies on the left side of the jaw [68]. The reasons for this difference in polar bear breakage between the canines is unknown but we speculate it could be associated with fighting behaviors and how males interact. In both sexes, the occurrence of scars was greater in individuals with breakage, providing a linkage between wounds and canine damage as coinciding injuries obtained in events of intraspecific fighting. Males had a higher proportion of individuals with both breakage and scars compared to females and our results support past studies [8,23] that suggested that these injuries were linked to male-male competition during the mating season. The most intense competition between adult males likely occurs in the prime breeding years between 11-17 years of age [34]. In support, we found similar rates of breakage between the sexes until males reached 11 years of age when breakage began to increase. Scarring and breakage in female polar bears also likely occurs from conspecific interactions, these events may be uncommon but could be associated with protection of young from infanticidal males [69] or protection of food resources [70]. Overall differences in mean breakage between the sexes suggest breakage is more strongly related to mate competition in males. Changes to the OSR may increase competition for mates [31]. Therefore, shifts towards a male-biased OSR could lead to increased occurrences of breakage and scarring. An additional possible mechanism of increased competition could lie in decreased habitat availability. Male polar bears reduce conflict during the mating season by sequestering estrous females away from other competitors [8,23]. Strategies of female sequestration can become ineffective under scenarios of increased population density [71]; therefore, greater instances of male-male competition and injury may occur if polar bear densities increase with reduced sea ice availability due to reduced effectiveness of traditional mating strategies. However, WH sea ice conditions during the mating season have likely not yet declined to a point where this mechanism would apply. Intersexual differences in foraging ecologies could also explain our breakage findings. Due to their larger size, male polar bears can hunt much bigger prey, such as bearded seals (*Erignathus barbatus*), than female counterparts who primarily consume ringed seals (*Pusa hispida*) [13,72]; attacking and handling these larger prey items may put greater stress on the canines which could result in more breakage [5]. However, understanding whether dietary differences influence sexually dimorphic traits is challenging because these characteristics cannot be easily disentangled from sexual selection pressures when determining a trait’s evolution [23]. Therefore, further research may be required to support or refute dietary niches as a possible mechanism of differing breakage rates between the sexes.

Overall, we found age- and sex-based differences in breakage and scarring of WH polar bears. While our study found some minor suggestions of a temporal component to breakage and scarring trends, we cannot determine whether there are increasing rates of injury or shifts in mating system competition in response to subpopulation structure changes. Continued monitoring and analysis of intraspecific injuries in the WH is needed for insights into how changes in behaviour associated with the mating system may be affected by altered population structures. Future research directions may include investigations of how canine breakage may affect survival. In addition, although challenging, research on predatory behaviour relative to canine breakage would provide new insights on the impacts of this injury.

## Supporting information

S1 Fig Sexual dimorphism in adult polar bear canines. Coloured arrows denote measurements taken on the specimen and inform robustness ratios. All measurements are in centimetres and arrows are not to scale. Purple arrows correspond to the canine length ratio (CLR =  length of canine/distance from inferior postorbital process to ridge of orbit) and the blue arrows in the inset images represent the canine thickness ratio (CTR =  width of canine tooth tip/width of canine tooth base). (A) Adult male skull; CLR: 5.2/2.8 =  1.9, CTR: 1.0/3.6 =  0.3. (B) Adult female skull; CLR: 4.7/2.7 =  1.7, CTR: 0.7/2.9 =  0.2. Images taken by Simonne S. Tremblay.
(TIF)

S2 Fig Additional examples of canine breakage in polar bears. (A) Canine classification (starting top left clockwise): 1, 3, 2, 3. (B) Canine classification (starting top left clockwise): 3, 3, 3, 3. Images taken by David McGeachy.
(TIF)

S1 File Temporal trend analysis using only adult bears. (PDF)
