# Supplementary figures and images for: Patterns and temporal trends in canine breakage and scarring in Western Hudson Bay polar bears (Ursus maritimus)
Source: PLoS One. 2025 Mar 25;20(3):e0319753. doi: 10.1371/journal.pone.0319753 (PMC11936232; doi:10.1371/journal.pone.0319753)

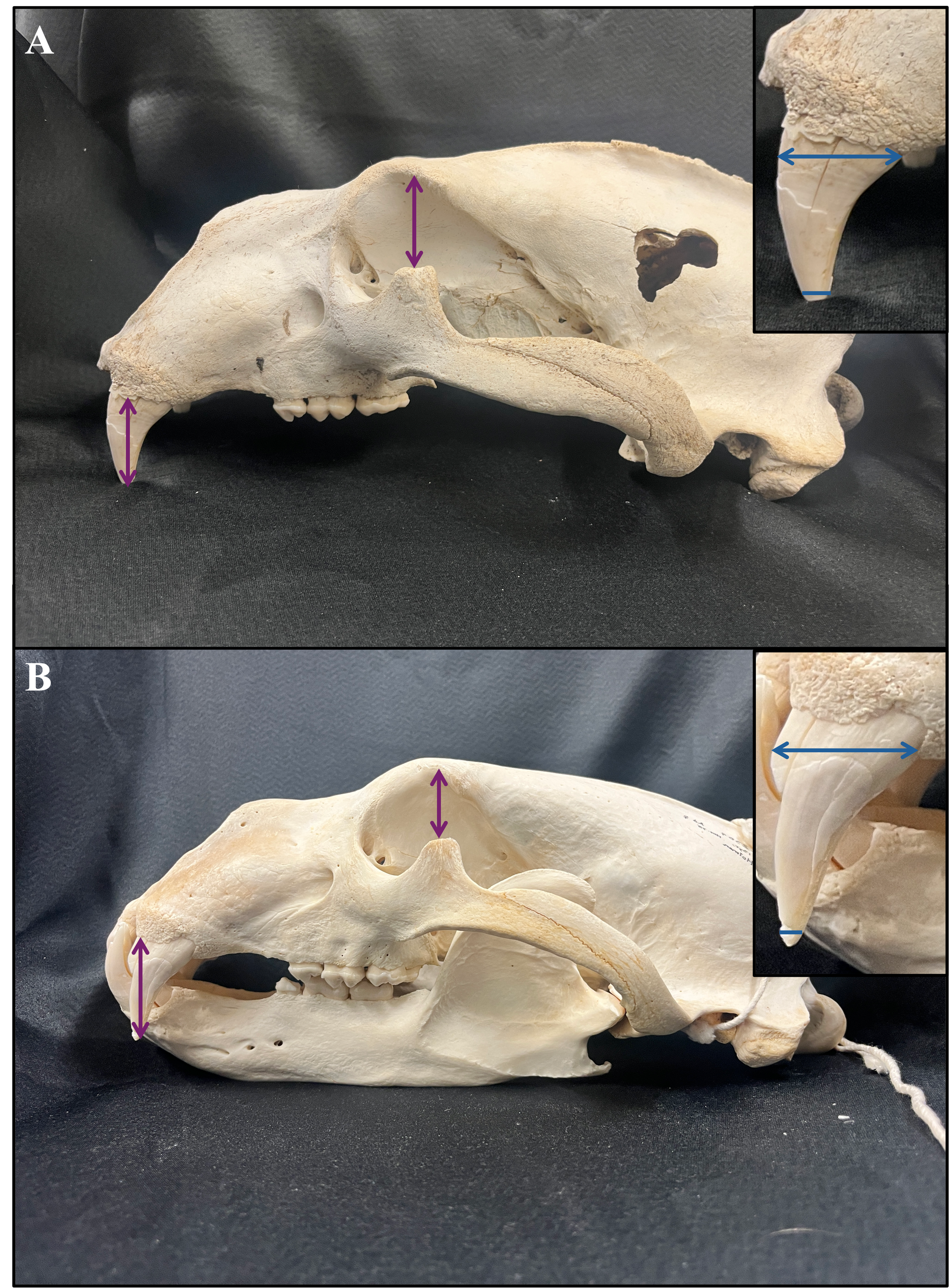

Supplement: S1 Fig — Coloured arrows denote measurements taken on the specimen and inform robustness ratios. All measurements are in centimetres and arrows are not to scale. Purple arrows correspond to the canine length ratio (CLR = length of canine/distance from inferior postorbital process to ridge of orbit) and the blue arrows in the inset images represent the canine thickness ratio (CTR = width of canine tooth tip/width of canine tooth base). (A) Adult male skull; CLR: 5.2/2.8 = 1.9, CTR: 1.0/3.6 = 0.3. (B) Adult female skull; CLR: 4.7/2.7 = 1.7, CTR: 0.7/2.9 = 0.2. Images taken by Simonne S. Tremblay. (TIF) [file pone.0319753.s001.tif]

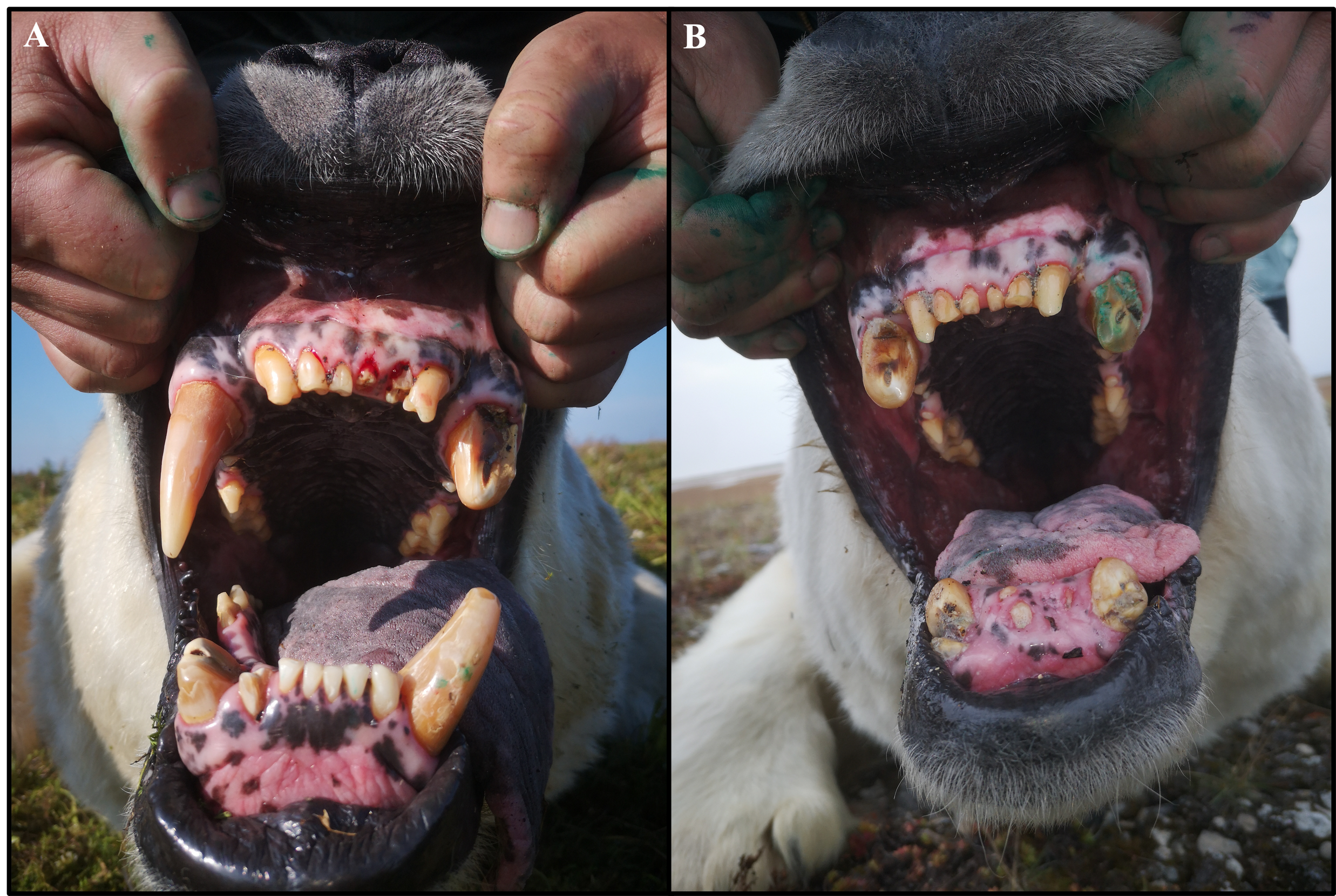

Supplement: S2 Fig — (A) Canine classification (starting top left clockwise): 1, 3, 2, 3. (B) Canine classification (starting top left clockwise): 3, 3, 3, 3. Images taken by David McGeachy. (TIF) [file pone.0319753.s002.tif]
