# Supplementary material for: Patterns and temporal trends in canine breakage and scarring in Western Hudson Bay polar bears (Ursus maritimus)
Source: PLoS One. 2025 Mar 25;20(3):e0319753. doi: 10.1371/journal.pone.0319753 (PMC11936232; doi:10.1371/journal.pone.0319753)
Supplement: S1 File — (PDF) [file pone.0319753.s003.pdf]

S1 File – Temporal Trend Analysis Using Only Adult Bears

As the only predictor, year was significant when predicting mean breakage in adult males and found an increasing trend (linear mixed effect model: y = -7.293 + 0.0042year, $p_{year} < 0.0001$, conditional $R^2 = 0.343$, marginal $R^2 = 0.020$). Year became marginally significant when age was added into the model (linear mixed effect model: y = -2.556 + 0.0017year + 0.0329age, $p_{year} = 0.075$, $p_{age} < 0.0001$, conditional $R^2 = 0.491$, marginal $R^2 = 0.253$). However, only the interaction term was significant in the interactive model as year became insignificant and age was marginally significant (linear mixed effect model: y = 6.049 – 0.0026year – 0.6415age + 0.0003year*age, $p_{year} = 0.256$, $p_{age} = 0.055$, $p_{year*age} = 0.044$, conditional $R^2 = 0.492$, marginal $R^2 = 0.254$). Similarly, year was a significant predictor with an increasing trend in adult female mean breakage as the only fixed effect (linear mixed effect model: y = -1.016 + 0.0010year, $p_{year} = 0.010$, conditional $R^2 = 0.324$, marginal $R^2 = 0.006$) but lost significance in the additive model (linear mixed effect model: y = -0.2821 + 0.0006year + 0.0062age, $p_{year} = 0.108$, $p_{age} < 0.0001$, conditional $R^2 = 0.358$, marginal $R^2 = 0.066$). All three variables were significant when predicting adult female temporal mean breakage in the interactive model (linear mixed effect model: y = 6.735 – 0.0029year – 0.5352age – 0.0003year*age, $p_{year} = 0.001$, $p_{age} < 0.0001$, $p_{year*age} < 0.0001$, conditional $R^2 = 0.364$, marginal $R^2 = 0.078$). In the full model evaluation of adult mean breakage, the top model included the fixed predictors age and sex and the first order interactive term of age*sex (Table 1; conditional $R^2 = 0.483$, marginal $R^2 = 0.254$, standardised regression coefficient & 95% CI: Intercept = -0.20 [-0.25, -0.14], Age = 0.14 [0.10, 0.19], Sex = 0.55 [0.47, 0.64], Age*Sex = 0.60 [0.52, 0.67]).

Table 1. Top three linear mixed effect models for adult mean breakage prediction in western Hudson Bay polar bears based on Akaike information criterion (AIC).

Model Rank	Predictors[a]	Log-likelihood	AIC	ΔAIC
1	Age, Sex, Age*Sex	301.74	-591.50	0.00
2	Age, Sex, Year, Age*Sex,	297.61	-581.20	10.25
3	Age, Sex, Year, Age*Sex, Age*Year	297.12	-578.20	13.24

[a] Fixed effect predictors include age of individual, sex, and year, with the models testing main effects, first-order interactions between the main effects, and three-way interaction between main effects. Random effect predictor was individual.

For scarring temporal trends in adult bears, the top model for maximum scarring probabilities included all three main effects of age, sex, and year (multinomial logistic regression: rank 1_{age}: = 0.20, SE = 0.01, 95% CI = [0.17, 0.22], p < 0.0001; rank 2_{age}: = 0.31, SE = 0.02, 95% CI = [0.26, 0.35], p < 0.0001; rank 1_{sex}: = 3.52, SE = 0.20, 95% CI = [3.12, 3.92], p < 0.0001; rank 2_{sex}: = 4.35, SE = 0.36, 95% CI = [3.64, 5.05], p < 0.0001; rank 1_{year}: = 0.01, SE = 0.01, 95% CI = [0.003, 0.03], p = 0.014; rank 2_{year}: = -0.01, SE = 0.01, 95% CI = [-0.03, 0.009], p = NA) (Table 2). P-value estimates were omitted for coefficients with Hauck-Donner effects, such as the top model's $rank1_{intercept}$ and $rank2_{year}$.

Table 2. Top three mixed multinomial logistic regression models for adult maximum scar value prediction in western Hudson Bay polar bears from 1981-2023 using Akaike information criterion (AIC).

Model Rank	Predictors[a]	Log-likelihood	AIC	ΔAIC
1	Age, Sex, Year	-989.98	1995.96	0.00
2	Age, Sex	-994.94	2001.87	5.91
3	Sex, Year	-1174.95	2361.90	365.94

[a] Main fixed effect predictors include age of individual, sex, and year. Random effect predictor was individual.
